# Uneven Implementation of Nirsevimab Prophylaxis Resulted in Non-Uniform Reductions in RSV-Related Hospitalizations in Italy

**DOI:** 10.3390/idr17050115

**Published:** 2025-09-12

**Authors:** Melodie O. Aricò, Francesco Accomando, Daniela Trotta, Giulia Marozzi, Anthea Mariani, Claudia Rossini, Claudio Cafagno, Letizia Lorusso, Martina Fornaro, Enrico Valletta, Désirée Caselli, Maurizio Aricò

**Affiliations:** 1 Department of Pediatrics, G. B. Morgagni—L. Pierantoni Hospital, Azienda Unità Sanitaria Locale Romagna, 47121 Forlì, Italy; francesco.accomando@auslromagna.it (F.A.); enrico.valletta@auslromagna.it (E.V.); 2 School of Pediatrics, University of Bologna, 40138 Bologna, Italy; 3 Pediatrics, S. Spirito Hospital, Azienda Sanitaria Locale Pescara, 65124 Pescara, Italy; daniela.trotta@asl.pe.it (D.T.); anthea.mariani@asl.pe.it (A.M.); maurizio.arico55@gmail.com (M.A.); 4 Department of Pediatrics and Neonatology, Ospedale Generale Provinciale, Azienda Sanitaria Territoriale Macerata, 62100 Macerata, Italy; giulia.marozzi@sanita.marche.it (G.M.); martina.fornaro@sanita.marche.it (M.F.); 5 School of Pediatrics, University of Siena, 53100 Siena, Italy; 6 Pediatric Infectious Diseases, Children’s Hospital Giovanni XXIII, Azienda Ospedaliero Universitaria Consorziale Policlinico di Bari, 701243 Bari, Italy; c.rossini4@studenti.uniba.it (C.R.); claudio.cafagno@policlinico.ba.it (C.C.); desiree.caselli@policlinico.ba.it (D.C.); 7 School of Medical Statistics and Biometry, Interdisciplinary Department of Medicine, University of Bari Aldo Moro, 70124 Bari, Italy; l.lorusso80@studenti.uniba.it

**Keywords:** bronchiolitis, respiratory syncytial virus, nirsevimab, health disparities

## Abstract

Background/Objectives: Respiratory syncytial virus (RSV) bronchiolitis remains a leading cause of hospitalization in infants. In the 2024–2025 season, passive newborn immunization with nirsevimab, a long-acting anti-RSV monoclonal antibody, was introduced for the first time in Italy. However, the immunization campaign was not uniformly implemented on a regional basis due to supply and organizational difficulties. The aim of the study was to assess the real-world impact of nirsevimab prophylaxis during the 2024–2025 bronchiolitis season in four regions of Italy. Methods: This multicenter observational study included infants <12 months hospitalized for bronchiolitis across four Italian centers. Hospitalizations due to RSV and non-RSV bronchiolitis were compared across the 2023–2024 and 2024–2025 seasons, in relation to the timing and coverage of nirsevimab’s introduction in each of the four regions. Results: Early and widespread nirsevimab administration was associated with a significant reduction in RSV hospitalizations and severity of disease. Centers located in regions that had delayed implementation of immunization observed higher RSV burden and intensive care unit admissions. Admissions for non-RSV bronchiolitis remained stable. Conclusions: Timely and universal administration of nirsevimab significantly reduced RSV hospitalizations and severity, while delayed implementation resulted in limited benefit. These findings support early and uniform prophylaxis to mitigate health disparities and seasonal pressure on pediatric healthcare systems.

## 1. Introduction

Bronchiolitis is a clinical syndrome characterized by respiratory distress, primarily affecting infants during their first year of life. The disease typically begins with upper respiratory tract symptoms, such as rhinitis, and progresses to lower respiratory involvement. In otherwise healthy infants, bronchiolitis is usually a self-limiting illness and resolves without complications. Most mild cases can be managed at home with supportive care, including hydration, nasal clearance, and careful monitoring for clinical deterioration.

Despite this, bronchiolitis remains a leading cause of hospitalization among newborns and infants [1,2]. Infants born prematurely, or with underlying cardiopulmonary conditions or immunodeficiency, are at higher risk of complications, including respiratory failure and secondary infections. Furthermore, approximately 30% of previously healthy infants hospitalized with bronchiolitis may experience recurrent wheezing later in childhood [3].

Respiratory syncytial virus (RSV) is the most common etiologic agent of bronchiolitis. Other contributing pathogens include rhinovirus, influenza viruses, parainfluenza viruses, and Mycoplasma pneumoniae. Despite its high disease burden, RSV-related mortality among hospitalized children in high-income countries remains low, typically below 1 per 1000 cases [4,5].

The recent introduction of passive RSV immunization is reshaping the clinical and public health landscape of bronchiolitis. Nirsevimab is a recombinant human IgG1 kappa monoclonal antibody that binds the F1 and F2 subunits of the RSV fusion (F) protein at a highly conserved epitope and locks the RSV F protein in the prefusion conformation to block viral entry into the host cell [6]. The introduction of nirsevimab has shown significant effectiveness in reducing severe RSV bronchiolitis, emergency visits, and hospitalizations. In the Harmonie randomized study of 8057 infants enrolled between August 2022 and February 2023, at 180 days, only 12 (0.3%) of 4038 infants in the nirsevimab group versus 68 (1.7%) of 4019 infants in the standard care group had been hospitalized for RSV-associated lower respiratory tract infection, corresponding to a nirsevimab efficacy of 82.7% (*p* < 0.0001) [7]. In a very recent study of 31,900 infants, 15,647 (49.1%) received nirsevimab. There were 35 RSV lower respiratory tract disease (LRTD) episodes (6.10/1000 person-years) among nirsevimab-immunized infants vs. 462 (58.51/1000 person-years) among nonimmunized infants. Nirsevimab effectiveness was 87.2% against RSV LRTD, 98.0% against hospitalized RSV LRTD, and 71.0% against PCR-confirmed RSV [8]. Countries that implemented universal nirsevimab immunization in the 2023–2024 season—such as the United States, France, Spain, and Luxembourg—have reported encouraging results [6,7,8,9,10,11,12]. Passive immunization has the potential not only to improve clinical outcomes in infants—particularly those at the highest level of risk—but also to reduce the emotional, logistical, and financial burdens on families and society [13]. Moreover, by lessening the demand for hospital admissions and respiratory support, such programs alleviate seasonal pressure on pediatric healthcare systems. Over time, widespread coverage may also reduce post-bronchiolitis complications, such as recurrent wheezing and asthma, and healthcare costs.

In this study, we document the real-world impact on epidemiological and clinical outcomes of passive immunization with nirsevimab, which was introduced at varying times across four Italian regions for the first time in the 2024–2025 bronchiolitis season.

## 2. Materials and Methods

Study Design. This multicenter, observational study was conducted by our Bronchiolitis Collaborative Group [14,15] to evaluate the real-world effectiveness of nirsevimab prophylaxis during the 2024–2025 bronchiolitis season in Italy.

Setting. Data were collected from four pediatric centers: center 1 (Bari, Apulia region): Pediatric Infectious Diseases Unit, tertiary teaching hospital; center 2 (Forlì, Emilia-Romagna region); center 3 (Macerata, Marche region); and center 4 (Pescara, Abruzzo region). Pediatric wards were embedded in second-level general hospitals. These centers are in regions in which nirsevimab prophylaxis was introduced with different timings between September 2024 and January 2025 and with different policies for recall.

Study Population. Data were collected for all infants under 12 months of age, hospitalized for clinical bronchiolitis in the four participating centers between 1st November and 31st March of the years 2023–2024 (period 1) and 2024–2025 (period 2). Data collected included the following: age, gender, gestational age, neonatal risk factor (prematurity and cardiopulmonary disease), and nirsevimab administration (status and timing). Diagnostic confirmation of RSV or other respiratory pathogens followed local protocols using commercially available rapid antigen tests or PCR. The study was conducted in accordance with the guidelines of the Declaration of Helsinki. Patient consent was waived since data were anonymized.

Outcomes and Analysis. The primary outcome was the comparison of the number of infants hospitalized with bronchiolitis during 2023–2024 and 2024–2025 RSV seasons, stratified by month and immunization status. Data were analyzed separately by period and by center. Categorical variables were shown using frequencies and percentages, and comparisons were performed using the chi-squared test or Fisher’s exact test, as appropriate. Tukey’s test was used for pairwise multiple comparisons for frequencies. The Kolmogorov–Smirnov test assessed normality. Variables were summarized as mean ± SD if normally distributed, or median (IQR) otherwise. Mean comparisons used ANOVA or non-parametric tests when assumptions were not met. Continuous variables such as age, days of oxygen therapy administration, and length of hospital stay, were categorized based on tertiles or median values.

To compare periods within each center, non-normal continuous variables were rank-transformed and analyzed using a general linear model with time per center interaction, followed by Tukey–Kramer correction. For categorical variables, *p*-values were adjusted using the false discovery rate (FDR) method [16].

To reduce confounding, a propensity score model was applied using inverse probability of treatment weighting (IPTW). The weight was estimated using the average treatment effect (ATE) and used for subsequent outcome analyses [17]. Immunization status was modelled as the dependent variable, including infants’ weight (kg), neonatal risk factors, and gender as covariates. We applied propensity score trimming to mitigate extreme weight influence on variance and bias [18]. Covariate balance was assessed using absolute standardized mean differences (SMDs). For variables showing residual imbalance (SMD > 0.1) after IPTW, they were incorporated as covariates into the outcome model [19,20]. After selecting variables of interest, listwise deletion was applied, resulting in 459 observations [21,22,23]. Univariate and multivariable logistic regressions identified factors associated with RSV positivity. Variables with *p* < 0.25 in univariate analysis, along with age group and immunization status, were included in the multivariable model. Results are reported with crude and weighted odds ratios (ORs) with 95% confidence intervals (CIs). All analyses were conducted using SAS^®^ software version 9.4, considering a two-sided *p*-value < 0.05 as statistically significant. The graphs were created using R Software version 4.4.1 (R Foundation for statistical computing, Wien, Austria) [24] and Excel (Microsoft, Redmond, Washington, DC, USA).

## 3. Results

A total of 471 children were enrolled across four centers during the two epidemic seasons under evaluation. Immunization against RSV with nirsevimab was started according to different timings as follows: centers 1 (Southern Italy) and 2 (Northern Italy) initiated nirsevimab administration in November 2024; center 3 (Central Italy) initiated it in December 2024; and center 4 (Central Italy) started nirsevimab administration in January 2025.

The recall strategies for children born in 2024 before the immunization start date were not uniform: center 1 recalled all children born after 1st July, and center 2 recalled all children born after 1st September. Center 3 recalled all children born after 1st October, although, due to a shortage of the monoclonal antibody, children weighing less then 5 kg had priority. Starting from 1st January, children with risk factors were also immunized if they weighed more than 5 kg. Center 4, due to a shortage of the drug, could not recall children born before the start of the immunization.

**Figure A1:**
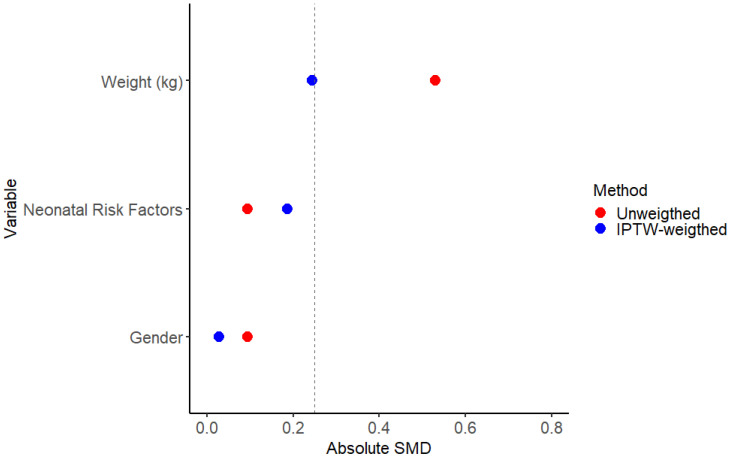
Absolute standardized mean differences (SMDs) in unweighted and weighted samples. The dotted line is set at a value of 0.25 for the absolute SMD.

**Figure A2:**
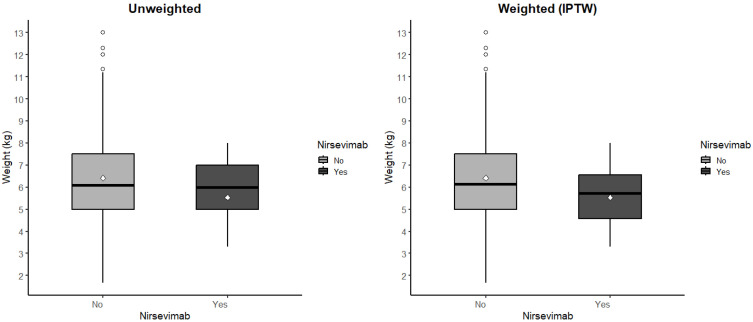
Boxplot comparison between unweighted and IPTW-weighted distributions. Outlier values are represented by circles, whereas the diamond in the center of the figure denotes the mean of the values shown.

Their main features, stratified by period, are summarized in Table 1. To reduce potential confounding across centers among the two periods, a propensity score-based model was applied using IPTW. The results of this analysis are described in detail in Appendix A (Figure A1 and Figure A2, Table A1, Table A2, Table A3, Table A4, Table A5 and Table A6).

### 3.1. Epidemiological Outcomes

**Figure 1:**
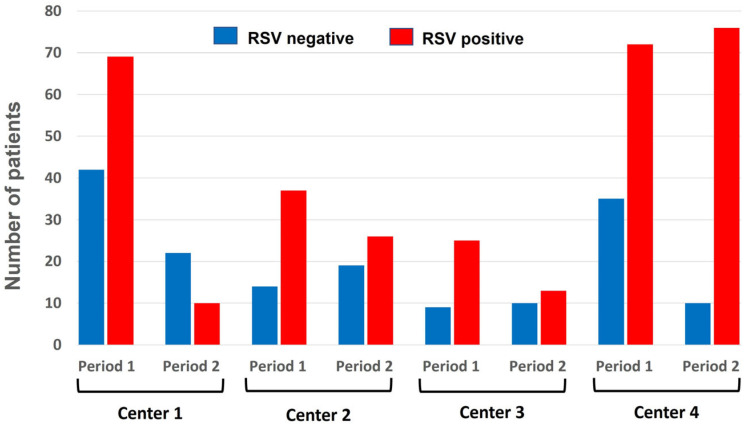
Proportion of RSV-positive (red column) versus RSV-negative (blue column) cases in 471 children up to 12 months old hospitalized for bronchiolitis by each center and across the two periods.

**Figure 2:**
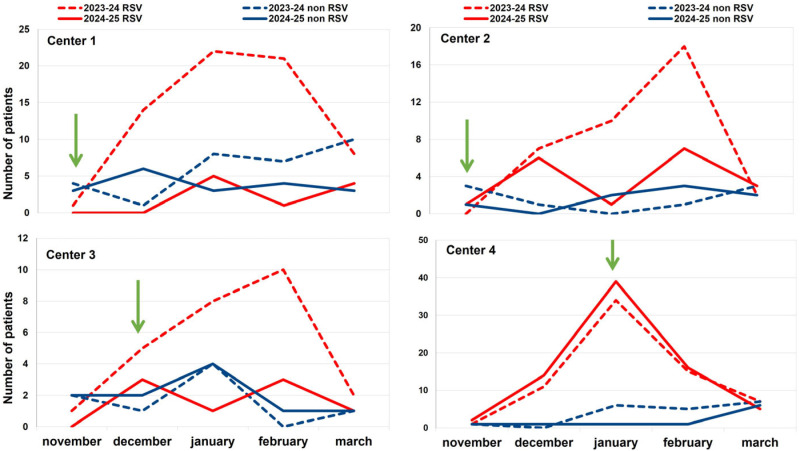
Comparison of the monthly distribution of hospital admissions for bronchiolitis in 471 children up to 12 months old in the four participating centers in 2023–2024 (dotted lines) and 2024–2025 (continuous lines) seasons, according to RSV status. Red lines show the trend of RSV-positive cases; the blue lines show the trend of the RSV-negative cases. The green arrow represents the time of the start of immunization.

As depicted in Figure 1, during the 2024–2025 season (period 2), after the introduction of nirsevimab, the number of admissions for bronchiolitis due to RSV in the four participating centers decreased significantly compared to the number observed during the previous season (period 1). The reduction was as follows: center 1 observed a reduction of 85% in admissions for RSV bronchiolitis (OR = 0.342 (95% CI [0.139, 0.844])), indicating a 66% lower odds of RSV positivity in the second period; in center 2, the reduction was 51% (OR = 0.542 (95% CI [0.206, 1.425])), corresponding to a 46% reduction in odds, but not statistically significant; in center 3, the reduction rate was 68% (OR = 0.288 (95% CI [0.087, 0.958])), reflecting a 71% decrease in odds. Otherwise, center 4 failed to achieve any reduction, since the number of observed cases increased by 9% in comparison with the 2023–2024 season. Figure 2 illustrates these data by showing the pattern of the monthly distribution of admissions for bronchiolitis across the four participating centers, which is also related to the timing of the start of the immunization program.

**Figure 3:**
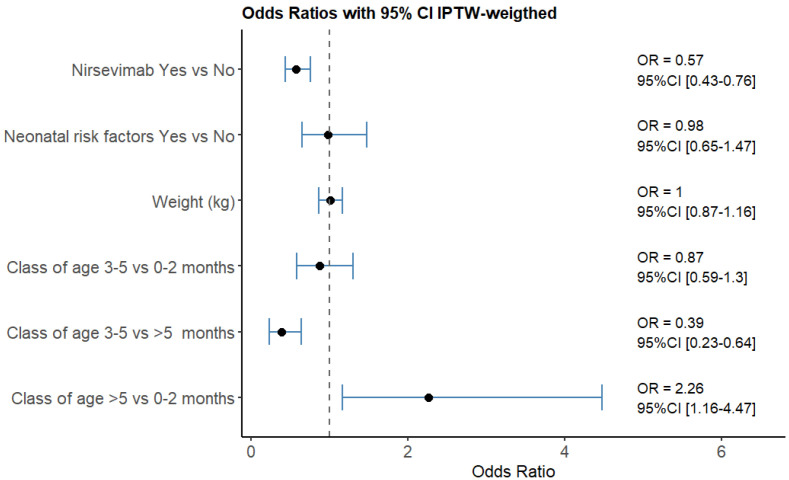
Results of the weighted multivariable logistic regression models. Patients older than 5 months were found to have a higher risk of contracting RSV compared to younger age groups.

Figure 3 shows the weighted multivariable regression results. Immunization significantly reduced RSV cases (OR = 0.57, 95% CI [0.43–0.76]), and infants’ weights and prenatal risk factors showed no significant difference, as expected. Patients older than 5 months were found to have a higher risk of contracting RSV compared to younger age groups.

Overall, during the 2024–2025 season, 23 children were hospitalized for bronchiolitis after having received passive immunization with nirsevimab. Of them, 12 tested positive for RSV, while 11 tested negative. The time between nirsevimab administration and admission for bronchiolitis in these 12 children ranged between 2 and 129 days, with a median time of 45 days.

### 3.2. Clinical Outcomes

In Appendix A Table A1, the main features of the patients admitted in the two periods are summarized by center of admission; in Appendix A Table A2, the main features of the patients are summarized by RSV status.

The proportion of children treated with HFNC decreased, although not significantly, in all centers timely starting immunization, but not in center 4.

During the 2024–2025 season, hospitalizations in the ICU increased (although not significantly) only in center 4, while no admissions to the ICU were registered in the other three centers.

It is interesting to note that in center 2, in which CPAP in the pediatric ward had previously been made available, 15 cases were treated with CPAP in 2023–2024, and only three (two of which were RSV-positive) in the season following nirsevimab administration (*p* = 0.01).

Of the 12 children admitted for RSV bronchiolitis despite nirsevimab prophylaxis, only a 2-month-old child who had received passive immunization two days prior to admission needed HFNC and intensive care treatment.

Table A2 illustrates the main features of children with bronchiolitis admitted during the 2024–2025 season compared with the previous season: the significantly more frequent use of HFNC in RSV+ cases reflects more aggressive disease in children who did not receive early immunization, and this was also associated with an extended duration of respiratory support by oxygen therapy.

## 4. Discussion

This multicenter observational study provides compelling real-world evidence that the early and widespread implementation of nirsevimab prophylaxis significantly reduces the clinical and epidemiological burden of RSV bronchiolitis in infants. Our findings align with international data [6,7,25,26] and emphasize the critical role of timely and coordinated immunization efforts.

In particular, centers 1 (Southern Italy) and 2 (Northern Italy), which initiated nirsevimab administration in November 2024, and center 3 (Central Italy), which initiated the program in December, experienced similar, substantial reductions, ranging from 51% to 85%, in RSV-related bronchiolitis hospitalizations among infants under 12 months. The effect of different recall strategies might contribute to these minor differences, which deserve further investigation. In contrast, center 4 (Central Italy), which delayed its campaign until January 2025, resulting in inferior protection against RSV bronchiolitis, failed to reduce the number of hospitalizations for bronchiolitis, which instead increased by 9%.

The early implementation of the immunization campaign was associated not only with fewer admissions but also with a milder clinical course of bronchiolitis. Centers with early rollout reported the reduced—though not statistically significant—use of HFNC, and notably, a sharp reduction in the number of children requiring CPAP in center 2, where such intervention is routinely available within the ward [27,28]. Meanwhile, center 4 recorded increased ICU admissions, underscoring the consequences of delayed protection.

A shift in RSV-related admissions toward older infants (>5 months) may reflect the effect of early immunization, as nirsevimab delays the peak risk window. Additionally, regional variations in immunization strategy, such as the broader age coverage in Apulia (center 1) compared to a more selective approach in Marche (center 3), likely influenced overall outcomes. The earlier immunization begins, the greater the protective benefit—supported by the duration of antibody efficacy [6,9].

Breakthrough RSV infections were rare and milder in immunized infants, confirming the protective role of nirsevimab via real-world data, consistent with large trials like HARMONIE [11] and surveillance reports from France and Spain [14,26]. While effective, residual disease remains, emphasizing the need for vigilance.

Importantly, our findings illustrate the implications of Italy’s decentralized public health governance. Asynchronous nirsevimab rollout led to regional disparities in RSV burden. Similar challenges have been reported in other European countries [10,11,12], reinforcing the importance of nationally coordinated strategies.

The economic cost of inconsistent implementation is substantial. In Italy, pediatric ICU care costs are estimated at approximately EUR 1650–1800 per day, while general pediatric ward stays average around EUR 700 per day [29]. Regions with late rollout likely experienced avoidable financial strain due to longer and more intensive hospitalizations.

This study’s strengths include its multicenter, real-world design and the advantage of comparing interregional implementation strategies. However, its limitations include its observational nature, the variability in RSV testing and adherence to treatment protocols, and potential confounders, mitigated using a propensity-score-weighted model.

## 5. Conclusions

In conclusion, the early and coordinated administration of nirsevimab significantly reduces RSV hospitalizations and disease severity in infants. Delayed implementation weakens these benefits and increases clinical and economic burdens. A unified national strategy is essential to ensure equitable protection and the efficient use of healthcare resources.

## Figures and Tables

**Table 1:** Main characteristics of 471 children (≤12 months) hospitalized for bronchiolitis across two time periods (2023–2024 vs. 2024–2025).

Variable	Period 1	Period 2	*p*-Value
	(n = 303)	(n = 168)	
**Gender**			
Male	180 (59%)	85 (51%)	0.06
**Age Category**			0.4
0–2 months	116 (38%)	63 (37%)	
3–5 months	105 (35%)	50 (30%)	
>5 months	82 (27%)	55 (33%)	
**Weight in kg**			
Median (IQR)	6 [5–7.4]	6.3 [5.1–7.9]	0.1
**Neonatal risk factors, n (%)**	48 (16%)	21 (12%)	0.3
**Low-flow nasal cannula, n (%)**	110 (36%)	54 (32%)	0.4
**High-flow nasal cannula (HFNC), n (%)**	149 (49%)	87 (52%)	0.6
**Continuous positive airway pressure (CPAP)**	15 (5%)	3 (2%)	**0.001** *
**RSV positivity, n (%)**	203 (67%)	112 (67%)	0.9
**ICU admission, n (%)**	9 (3%)	14 (8%)	**0.01**
**Days of oxygen therapy, n (%)**			
0–3 days	164 (54%)	73 (43%)	**0.03**
>3 days	139 (46%)	95 (56%)	
**Days of hospital stay, n (%)**			
0–5 days	151 (50%)	67 (40%)	**0.04**
>5 days	152 (50%)	101 (60%)	

The bold *p*-values are <0.05 and the numbers with an * were calculated with Fisher’s exact test.

## Data Availability

Data is contained within the article.

## Appendix A

**Table A1:** Socio-demographic and clinical differences in bronchiolitis cases across two periods and four centers.

	Center 1			Center 2			Center 3			Center 4		
	Period 1	Period 2	*p*	Period 1	Period 2	*p*	Period 1	Period 2	*p*	Period 1	Period 2	*p*
Variable	(N = 111)	(N = 32)		(N = 51)	(N = 32)		(N = 34)	(N = 18)		(N = 107)	(N = 86)	
**Gender**												
Male	61 (55%)	15 (47%)	0.6	32 (63%)	19 (59%)	0.8	22 (65%)	7 (39%)	0.3	65 (61%)	44 (51%)	0.4
**Age**												
Median [IQR]	3 [2–6]	5.5 [7–1]	0.8	4 [2–7]	4.5 [3–8]	0.9	2 [1–4]	2.5 [1–5]	1	3 [2–6]	3 [2–6]	0.9
**Age category**												
0–2 months	35 (31.5%)	9 (28%)	0.1	19 (37%)	6 (19%)	0.3	18 (53%)	9 (50%)	0.9 *	44 (41%)	39 (45%)	0.9
3–5 months	47 (42%)	7 (22%)		13 (25.5%)	13 (41%)		9 (26.5%)	6 (33%)		36 (34%)	24 (28%)	
>5 months	29 (26%)	16 (50%)		19 (37%)	13 (41%)		7 (21%)	3 (17%)		27 (25%)	23 (27%)	
**Weight in kg**												
Median (IQR)	6 [5–7]	6.5 [8–3]	0.9	6.6 [5–8.29]	6.8 [5.9–7.78]	1	5.25 [4.3–7]	5.87 [4.21–7.55]	1	6.17 [4.99–7.5]	6.23 [5.1–7.6]	0.9
**RSV status**												
Positive	69 (62%)	10 (31%)	**0.004**	37 (72%)	18 (56%)	0.1	25 (73%)	8 (44%)	0.1	72 (67%)	76 (88%)	**0.002**
**Neonatal risk factors, n (%)**	19 (17%)	11 (34%)	0.07	4 (8%)	6 (19%)	0.2 *	7 (21%)	1 (6%)	0.2 *	18 (17%)	3 (3.5%)	**0.02 ***
**Low-flow nasal cannula, n (%)**	77 (70%)	15 (47%)	**0.04**	7 (14%)	10 (31%)	0.4	17 (50%)	11 (61%)	0.07	9 (8%)	18 (21%)	**0.04**
**High-flow nasal cannula (HFNC), n (%)**	27 (24%)	4 (12.5%)	0.4 *	21 (41%)	9 (28%)	0.4	6 (18%)	2 (11%)	0.7 *	95 (88%)	27 (83%)	0.4
**ICU admission**	0 (0%)	0 (0%)	n.c.	1 (2%)	0	1 *	1 (3%)	0	1 *	7 (6.5%)	14 (16%)	0.1
**Duration of oxygen therapy**												
0–3 days	46 (41%)	23 (72%)	**0.01**	34 (67%)	28 (87.5%)	0.08 *	27 (79%)	14 (78%)	1 *	32 (30%)	30 (35%)	0.6
>3 days	65 (59%)	9 (28%)		17 (33%)	4 (12.5%)		7 (21%)	4 (22%)		75 (70%)	56 (65%)	
**Hospital stays**												
0–5 days	75 (68%)	13 (41%)	**0.02**	14 (27%)	5 (16%)	0.6	9 (26.5%)	6 (33%)	0.8	53 (49.5%)	43 (50%)	0.9
>5 days	36 (32%)	19 (59%)		37 (73%)	27 (84%)		25 (73.5%)	12 (67%)		54 (50.5%)	43 (50%)	
**Nirsevimab**	-	8 (25%)		-	9 (28%)		-	3 (17%)		-	3 (3.5%)	

Bolded *p*-values indicate statistical significance (*p* < 0.05). * Fisher’s exact test was used when appropriate. n.c.: not countable.

**Table A2:** Comparison of the main features of 471 children with bronchiolitis admitted during the years 2023–2024 (period 1) or 2024–2025 (period 2), according to RSV status.

	Period 1			Period 2		
	RSV-Negative	RSV-Positive	*p*	RSV-Negative	RSV-Positive	*p*
	(N = 100)	(N = 203)		(N = 56)	(N = 112)	
**Gender**	55 (55%)	125 (62%)	0.7	28 (50%)	57 (51%)	0.99
Male						
**Age category**						
0–2 months	35 (35%)	81 (40%)	0.7	19 (34%)	44 (39%)	0.8
3–5 months	37 (37%)	68 (33.5%)		18 (32%)	32 (29%)	
>5 months	28 (28%)	54 (27%)		19 (34%)	36 (32%)	
**Weight in kg**						
Median [IQR]	6 [5–7]	6 [5–7.5]	0.99	6.035 [4.5–7.4]	6.52 [5.425–8]	**0.02**
**Neonatal risk factors. n (%)**	20 (20%)	28 (14%)	0.5	13 (23%)	8 (7%)	**0.03**
**Low-flow nasal cannula, n (%)**	34 (34%)	76 (37%)	0.9	17 (30%)	37 (33%)	0.99
**High-flow nasal cannula (HFNC), n (%)**	42 (42%)	107 (53%)	0.3	19 (34%)	68 (61%)	**0.007**
**Continuous Positive Airway Pressure (CPAP)**	2 (2%)	13 (6%)	0.5 *	1 (2%)	2 (2%)	1 *
**ICU admission**	2 (2%)	7 (3%)	0.9 *	1 (2%)	13 (12%)	0.2 *
**Duration of oxygen therapy**						
0–3 days	50 (50%)	89 (44%)	0.7	41 (73%)	54 (48%)	**0.01**
>3 days	50 (50%)	114 (56%)		15 (27%)	58 (52%)	
**Hospital stays**						
0–5 days	57 (57%)	95 (47%)	0.3	38 (68%)	63 (56%)	0.5
>5 days	43 (43%)	108 (53%)		18 (32%)	49 (44%)	
**Center**						
1	42 (42%)	69 (34%)	0.5	22 (39%)	10 (9%)	**<0.0001**
2	14 (14%)	37 (18%)		14 (25%)	18 (16%)	
3	9 (9%)	25 (12%)		10 (18%)	8 (7%)	
4	35 (35%)	72 (35%)		10 (18%)	76 (68%)	
**Nirsevimab**	-	-		11 (20%)	12 (11%)	

Bolded *p*-values indicate statistical significance (*p* < 0.05). * Fisher’s exact test was used when appropriate.

In Figure A1, the distribution of absolute standardized mean differences between the immunized and non-immunized infants of the considered covariates is shown. After the IPTW adjustment, the infants’ variables between the groups were well balanced, reaching an SMD < 0.1 for neonatal risk factors and gender. The absolute SMD weighted value for the infants’ weight variable is 0.24. The center variable was excluded, as its inclusion reduced the homogeneity of the propensity score distribution, potentially compromising the balance between treatment groups.

**Table A3:** Baseline percentage of gender between immunization group before and after IPTW weight.

	Unweighted n (%)			IPTW-Weighted %		
	Nirsevimab Immunization			Nirsevimab Immunization		
	No	Yes	*p*-Value	No	Yes	*p*-Value
	(N = 436)	(N = 23)		(N = 574)	(N = 23)	
**Gender**						
Female	188 (43%)	11 (48%)	0.6571	43%	42%	0.6781
Male	248 (57%)	12 (52%)		57%	58%	
**Weight in kg**						
Mean (SD)	6.41 (1.90)	5.54 (1.35)		6.37 (1.94)	5.97 (5.72)	
Median [Min, Max]	6.15 [5.0–7.5]	5.72 [4.4–6.6]	0.0569	6.1 [5.0–7.5]	6.0 [5.0–7.0]	**0.0311**
**Neonatal Risk Factors**						
No	375 (86%)	19 (83%)	0.5512 *	86%	79%	**0.0076**
Yes	61 (14%)	4 (17%)		14%	21%	

Bold *p*-values are < 0.05. * The *p*-value was calculated using Fisher’s exact test.

The infants’ baseline characteristics before and after IPTW are presented in Table A3. No differences in gender are displayed before IPTW (percentage of females: 43.1% vs. 47.8%; *p* = 0.6571) or after IPTW (43.35% vs. 41.98%; *p* = 0.6781). The prevalence of neonatal risk factors before IPTW (86.0% vs. 82.6%; *p* = 0.5512) and after IPTW (85.82% vs. 79.03%; *p* = 0.0076) was comparable, but after IPTW, the difference was statistically significant. The mean and median of the weight values were comparable before IPTW (6.41 (1.90) vs. 5.54 (1.35); *p* = 0.0569) and after IPTW (6.37 (1.94) vs. 5.97 (5.72); *p* = 0.0311), but the difference after IPTW was statistically significant. The increase in the standard deviation suggests a potential bias in the estimation, indicating greater variability among infants who received the nirsevimab immunization. In Figure A2, the distribution of the infants’ weight is shown for immunized and non-immunized infants, under both the unweighted and the IPTW-weighted models. To mitigate this bias, the two imbalanced variables were included in the outcome model.

The three tables (from Table A4, Table A5 and Table A6) present distinct multivariable logistic regression models.

The initial model (Table A4) excludes variables contributing to the propensity score calculation. Key differences from the final model include a statistically significant increase in RSV prevalence among infants >5 months compared to 0–2-month-olds (adjusted OR = 2.262, 95% CI [1.154–4.433]). The second model (Table A5) maintains this structure while incorporating infants’ weight as an additional covariate, demonstrating consistent effect estimates (adjusted OR = 2.236, 95% CI [1.182–4.229]). In the final model (Table A6), neonatal risk factor status was introduced as a stratification variable, which maintains the previously observed age-related significance, but lost significance from the unweighted to the weighted model (from OR = 0.528, 95% CI [0.307–0.911] to OR = 0.978, 95%CI [0.652–1.468]).

**Table A4:** Comparison of weighted and unweighted multivariable logistic regression models—Model1.

	Multivariable Logistic Analysis—Unweighted		Multivariable Logistic Analysis IPTW—Weighted	
Variable	OR	95%CI	OR Weighted	95%CI Weighted
**Nirsevimab: Yes vs. No**	0.486	[0.208–1.137]	0.572	[0.433–0.756]
**Age category: 3–5 months vs. >5 months**	0.912	[0.552–1.505]	0.386	[0.256–0.581]
**Age category: 3–5 months vs. 0–2 months**	0.829	[0.521–1.321]	0.879	[0.65–1.189]
**Age category: >5 months vs. 0–2 months**	0.910	[0.555–1.491]	2.277	[1.52–3.413]

**Table A5:** Comparison of weighted and unweighted multivariable logistic regression models—Model2.

	Multivariable Logistic Analysis —Unweighted		Multivariable Logistic Analysis IPTW—Weighted	
Variable	OR	95%CI	OR Weighted	95%CI Weighted
**Nirsevimab: Yes vs. No**	0.499	[0.213–1.169]	0.572	[0.433–0.756]
**Age category: 3–5 months vs. >5 months**	1.229	[0.694–2.175]	0.389	[0.243–0.625]
**Age category: 3–5 months vs. 0–2 months**	0.608	[0.352–1.052]	0.870	[0.586–1.292]
**Age category: >5 months vs. 0–2 months**	0.495	[0.235–1.042]	2.236	[1.182–4.229]
**Weight (kg)**	1.195	[1.014–1.408]	1.005	[0.868–1.164]

**Table A6:** Comparison of weighted and unweighted multivariable logistic regression models—Model3.

	Multivariable Logistic Analysis —Unweighted		Multivariable Logistic Analysis IPTW—Weighted	
Variable	OR	95%CI	OR Weighted	95%CI Weighted
**Nirsevimab: Yes vs. No**	0.510	[0.217–1.203]	0.573	[0.433–0.759]
**Neonatal risk factor: Yes vs. No**	0.528	[0.307–0.911]	0.978	[0.652–1.468]
**Age category: 3–5 months vs. >5 months**	1.179	[0.663–2.098]	0.386	[0.234–0.637]
**Age category: 3–5 months vs. 0–2 months**	0.663	[0.381–1.156]	0.873	[0.586–1.301]
**Age category: >5 months vs. 0–2 months**	0.562	[0.264–1.196]	2.262	[1.154–4.433]
**Weight (kg)**	1.173	[0.994–1.384]	1.005	[0.867–1.164]
